# Initial Presentation of Ovarian Carcinosarcoma With Non-islet Cell Tumor Hypoglycemia: A Case Report

**DOI:** 10.7759/cureus.65825

**Published:** 2024-07-31

**Authors:** Shatha Elemian, Samer Jumean, Amy Paige, Shahd Yaghi, Hamid S Shaaban

**Affiliations:** 1 Department of Internal Medicine, Saint Michael’s Medical Center, New York Medical College, Newark, USA; 2 Department of Pulmonary and Critical Care Medicine, Saint Michael's Medical Center, Newark, USA; 3 Department of Hematology and Oncology, Saint Michael's Medical Center, Newark, USA

**Keywords:** paraneoplastic syndromes, hypoglycemia, non-islet cell tumor, insulin-like growth factor-ii, ovarian carcinosarcoma

## Abstract

Ovarian cancer, although not among the most commonly diagnosed cancers, remains a significant cause of cancer-related mortality in females. Several paraneoplastic syndromes have been associated, and this case study represents a rare manifestation of ovarian cancer, presenting as non-islet cell tumor hypoglycemia (NICTH), characterized by the excessive production of insulin-like growth factor-II (IGF-II) by tumor cells. We report a 55-year-old woman who presented to our hospital with abdominal distension and severe refractory hypoglycemia. The laboratory data revealed the suppression of serum insulin and C-peptide levels. The insulin-like growth factor II (IGF-II)/insulin-like growth factor 1 (IGF1) ratio was >32. The hypoglycemia was hence attributed to the non-islet cell tumor type, and it is likely driven by tumoral secretion of incompletely processed IGF-II. The lab findings suggested the existence of NICTH. Abdominal computed tomography demonstrated the presence of a left ovarian mass and peritoneal carcinomatosis. CT-guided biopsy of the peritoneal lesions showed poorly differentiated malignancy consistent with ovarian carcinosarcoma (OCS). The patient was treated with a continuous infusion of glucose. She even received oral prednisone and glucagon infusion. Chemotherapy with carboplatin and paclitaxel was initiated, but unfortunately, she died from complications of multiorgan failure. To our knowledge, this is the first novel case of an initial presentation of metastatic OCS with NICTH, underscoring the complexity of ovarian cancer presentations and the necessity of a comprehensive approach in managing rare paraneoplastic syndromes, such as NICTH.

## Introduction

Ovarian cancer ranks as the eighth most commonly diagnosed cancer, yet it stands as the fifth leading cause of cancer-related death among women in the United States [1]. In 2023, the estimated incidence and death rates were recorded at 19,710 and 13,270, respectively [1]. Ovarian cancer can be classified based on the origin of tumor cells into epithelial, which account for 90% of all cases, followed by sex cord-stromal tumors and germ cell tumors. Ovarian carcinosarcoma (OCS), previously also known as mixed malignant Müllerian tumor, is an uncommon and highly aggressive type of ovarian cancer. It is considered to be biphasic, comprising malignant epithelial and malignant mesenchymal populations. Despite its very aggressive behavior, OCS has received relatively little research attention to date.

Ovarian tumors have been associated with several paraneoplastic syndromes. These syndromes can arise from the tumor's secretion of cytokines and hormones or may arise from an autoimmune response triggered by tumor cells against normal body cells [2]. Non-islet cell tumor hypoglycemia (NICTH) is an extremely rare paraneoplastic syndrome attributed to the excessive production of high molecular weight insulin-like growth factor (IGF-II) by tumor cells [3]. To our knowledge, there have only been two published case reports of NICTH in patients with recurrent carcinosarcoma of the uterus and ovary, respectively [4,5]. In this report, we describe the first case of NICTH as an initial presentation of OCS with peritoneal carcinomatosis.

## Case presentation

A 55-year-old female with a past medical history of hypertension and breast cancer, treated with bilateral mastectomy without radiation or chemotherapy six years ago, presented to the emergency department (ED) with abdominal distension associated with progressive worsening of shortness of breath upon exertion along with vaginal bleeding. Laboratory workup revealed a hemoglobin of 6.6 g/dL, mean corpuscular volume (MCV) of 70.1 fL, red cell distribution width (RDW) of 17.5%, and platelet count of 637 x 10^9^/L. Her international normalized ratio (INR) was 1.37, partial thromboplastin time (PTT) was 28.4 seconds, and prothrombin time (PT) was 14 seconds. In addition, the patient had a cancer antigen 125 (CA 125) of 252 (Table 1).

**Table 1:** Laboratory workup results with normal ranges INR: international normalized ratio, PTT: partial thromboplastin time, PT: prothrombin time, CA 125: cancer antigen 125, IGF: insulin-like growth factor

Lab test	Results	Normal range
Hemoglobin	6.6 g/dL	12-15 g/dl
Mean corpuscular volume	70.1 fL	80-99 fL
Red cell distribution width	17.5%	11-15%
Platelets	637 x 10^9^/L	150-450^9^/L
INR	1.37	0.9-1.1
PTT	28.4 seconds	26-35 seconds
PT	14 seconds	10-13 seconds
CA 125	252 U/ml	0-45 U/ml
Insulin	<0.4 μU/mL	2.6-24 4 μU/mL
C-peptide	ng/mL	1.1-4.4 ng/mL
Sulfonylurea screen	Negative	
IGF-I	26 ng/mL	65-216 ng/mL
IGF-II	842 ng/mL	333-967 ng/mL

During her hospitalization, the patient experienced multiple episodes of symptomatic hypoglycemia, with the lowest blood glucose level being 24 mg/dL. Insulin levels were <0.4 μU/mL, C-peptide was 0.1 ng/mL, a sulfonylurea screen was negative, IGF-I was 26 and IGF-II 842, and the IGFII/IGF1 ratio was >32. The hypoglycemia was attributed to the non-islet cell tumor type, likely driven by the tumoral secretion of incompletely processed IGF-II, affecting gluconeogenesis and glucose utilization in the liver. The patient was started on oral prednisone to decrease the amount of pro-IGF2, was kept on continuous dextrose 5%, and received a glucagon infusion.

**Figure 1:**
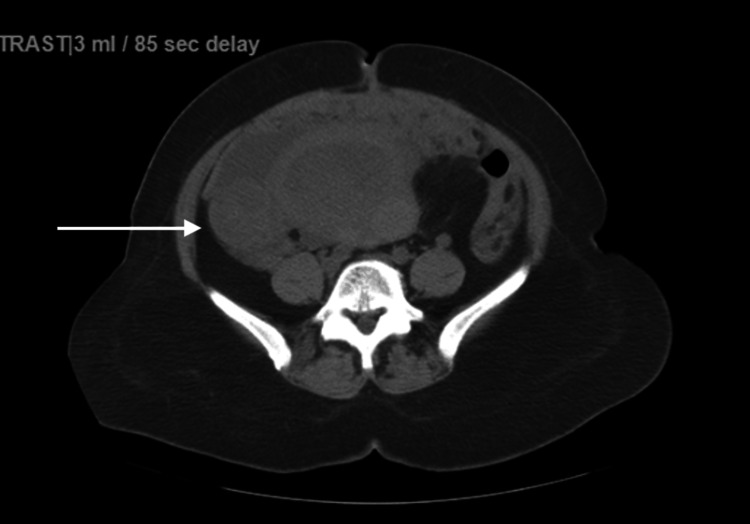
Abdominal computed tomography scan without enhanced contrast shows an enlarged uterus with a right adnexal mass measuring up 4.6 cm.

**Figure 2:**
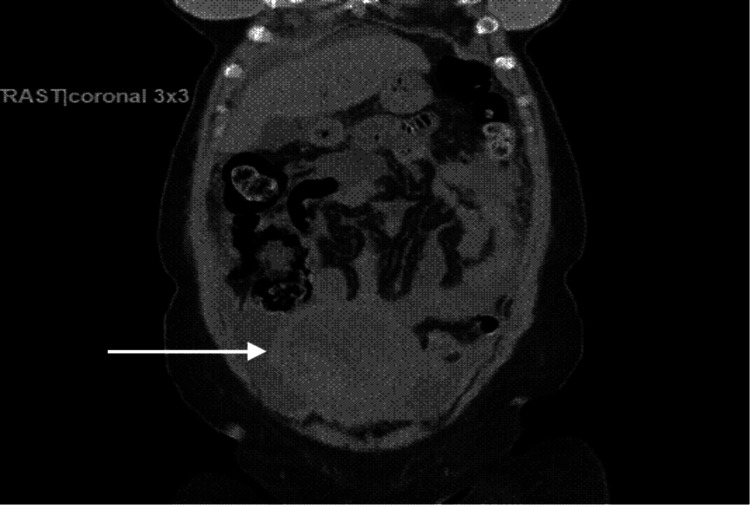
Coronal section of abdomen computed tomography scan without enhanced contrast shows an enlarged heterogeneous uterus with a small to moderate amount of ascites.

A transabdominal and transvaginal ultrasound showed an enlarged uterus measuring 15.3 x 7 x 8.6 cm with fibroids and a right ovary mass with a 5.8 x 3 x 4.5 cm mass with heterogeneous echotexture. A CT abdomen-pelvis without contrast revealed findings suspicious for ovarian neoplasm with associated peritoneal carcinomatosis and an enlarged uterus with several fibroids (Figures 1, 2). The patient received two units of packed red blood cells and her vaginal bleeding stopped.

**Figure 3:**
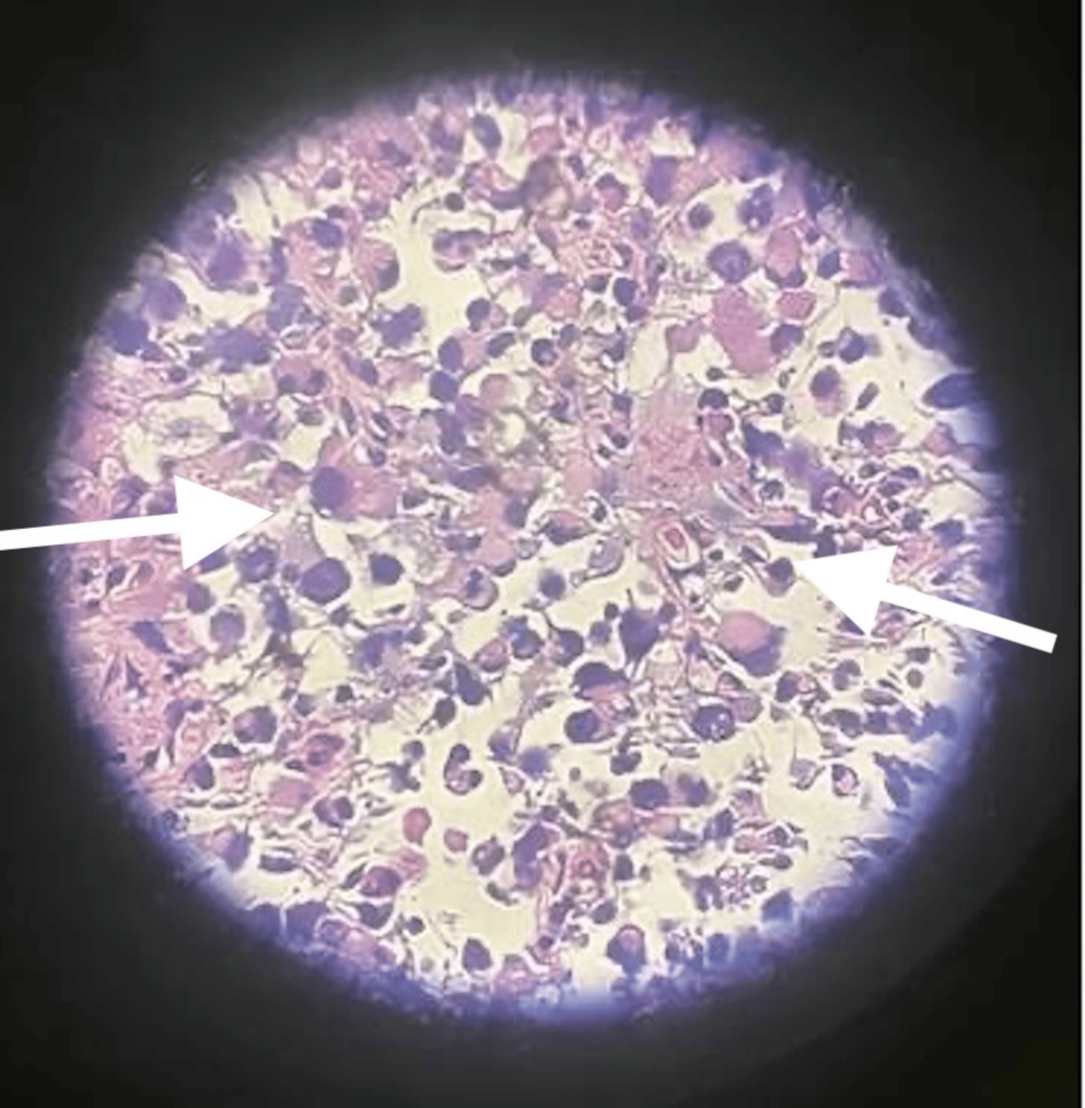
The histopathological finding demonstrates poorly differentiated malignancy consistent with sarcomatoid changes of carcinoma.

She subsequently had a CT-guided biopsy of the peritoneal lesions, and histopathology showed a poorly differentiated malignancy consistent with sarcoma/sarcomatoid changes of carcinoma (Figure 3). The tumor showed sheets of discohesive atypical cells with rhabdoid features and prominent nuclei. Immunohistochemical stains were positive for PAX8, pancytokeratin, and myoD1 and negative for Mum1, S100, CD30, ER, and GATA3. SMARCA4 stain showed intact nuclear expression, and the tumor exhibited a mutant expression pattern of p53. These histopathological findings favored the diagnosis of OCS.

She received one dose of carboplatin and paclitaxel to decrease tumor size and its paraneoplastic effects. However, two days post-chemotherapy, she developed multiorgan failure due to disease progression and unfortunately died.

## Discussion

NICTH is a condition in which hypoglycemia is induced by any neoplasm other than an insulinoma. In nearly all cases, the underlying mechanism of hypoglycemia in individuals with this syndrome results from the tumor's excessive production of insulin-like growth factor-II (IGF-II) [3]. Normally, circulating IGFs are bound to insulin growth factor-binding proteins (IGFBPs), which regulate their effects and reduce their availability [3]; however, studies have found that IGF-II that is produced by tumor cells is a partially processed IGF-II, referred to as big IGF-II due to incomplete glycosylation of precursor molecules [3]. This big IGF-II has diminished affinity to IGFBD and increased affinity to insulin receptors, which promotes hypoglycemia by enhancing peripheral glucose uptake. 

Historically, NICTH has been linked to various malignant and non-malignant tumors of mesenchymal or epithelial origin. Fukuda et al. [6] reviewed 78 patients with NICTH, identifying hepatocellular carcinoma as the most frequent cause, followed by gastric carcinoma and mesothelioma. In ovarian cases, NICTH has been documented in three instances: the first involving a 72-year-old woman with recurrent ovarian solitary fibrous tumor [7], the second concerning a 27-year-old woman presenting with metastatic ovarian yolk sac tumor, and the last case being a rare recurrent carcinosarcoma of the ovary [4].

OCS is a rare neoplasm, comprising just 4% of ovarian malignancies [8]. Typically found in postmenopausal women with a history of low parity, this aggressive sarcoma often manifests in advanced stages with a median overall survival time of 12 months [8]. Its aggressive nature is linked to increased levels of vascular endothelial growth factor (VEGF), a potent angiogenic factor, and an increased presence of small vessels, which are significant predictors of poor survival. Moreover, elevated expression of the mutated variant of the tumor suppressor protein p53 is commonly observed in gynecological cancers, including OCS, as was evidenced in our case.

NICTH typically presents with spontaneous hypoglycemia, resembling the hypoglycemia seen in functioning islet-cell tumors, but without elevated insulin levels [6]. However, symptoms related to the tumor mass effect may also precede hypoglycemia onset. In addition, some patients may present with hypokalemia, which occurs secondary to the insulin-like intracellular effects, and in rare cases [9], patients may exhibit acromegaloid features in the absence of elevated GH or IGF-1 due to the stimulation of IGF-II stimulation of IGF receptors [9]. NICTH and abdominal distension secondary to peritoneal carcinomatosis were the initial presentations of OCS in our patient.

Diagnosing NICTH necessitates a comprehensive approach that considers the patient's initial symptoms, tumor type, and recurrent hypoglycemia in the absence of organ damage, hormonal deficiencies, or medication-induced hypoglycemia. Laboratory findings will typically demonstrate low glucose levels concurrent with decreased levels of insulin, proinsulin, C-peptide, and β-hydroxybutyrate. There is also a concomitant negative oral hypoglycemic agent screen. Growth hormone (GH) levels tend to be low, unlike transient hypoglycemic episodes that elicit an increase in GH. Even if IGF-II levels appear normal, IGF-I levels are typically suppressed to less than 100 ng/mL, resulting in an elevated IGF-II:IGF-I ratio [10]. This ratio serves as a potentially valuable screening tool for NICTH in cases of hypoglycemia.

The long-term treatment strategies for NICTH primarily focus on complete tumor removal or reduction of tumor mass, which can reverse the metabolic alterations associated with NICTH. However, in cases where curative resection is not feasible due to extensive disease or metastasis, managing hypoglycemia becomes a therapeutic challenge. Various approaches have been attempted to relieve hypoglycemic symptoms [11], including chemotherapy, selective embolization of tumor mass, and increasing serum glucose levels through parenteral glucose administration or dietary adjustments. In addition, glucagon administration has shown short-term benefits in alleviating hypoglycemia. Somatostatin analogs like octreotide have been used [12], but their effectiveness in restoring glucose levels is generally limited [13]. Glucocorticosteroids have recently emerged as one of the most effective long-term treatments [14], stimulating gluconeogenesis and suppressing "big"-IGF-II production, although their efficacy may vary. GH therapy has also been explored, with mixed results in increasing insulin-like growth factor levels.

## Conclusions

Our case is the first case of an initial presentation of OCS with NICTH. The initial laboratory evaluation includes the measurement of glucose, insulin, proinsulin, and C-peptide during an episode of hypoglycemia. In contrast to the biochemical findings in individuals with hyperinsulinemic hypoglycemia, patients with IGF-induced hypoglycemia typically demonstrate depressed levels of serum insulin and C-peptide during hypoglycemia The diagnosis is usually confirmed by elevated IGF-2/IGF-1 ratios greater than 10:1. If the carcinosarcoma diagnosis is made at an early stage, complete resection can often cure hypoglycemia. However, our case highlights that severe refractory hypoglycemia persists especially when this extremely aggressive malignancy is initially diagnosed in the advanced stage with a high tumor burden. NICTH in these circumstances may possibly be a marker of poor prognosis as evidenced in our case.
